# Fuzzy Logic for Incidence Geometry

**DOI:** 10.1155/2016/9057263

**Published:** 2016-08-29

**Authors:** Alex Tserkovny

**Affiliations:** Dassault Systemes, 175 Wyman Street, Waltham, MA 02451, USA

## Abstract

The paper presents a mathematical framework for approximate geometric reasoning with extended objects in the context of Geography, in which all entities and their relationships are described by human language. These entities could be labelled by commonly used names of landmarks, water areas, and so forth. Unlike single points that are given in Cartesian coordinates, these geographic entities are extended in space and often loosely defined, but people easily perform spatial reasoning with extended geographic objects “as if they were points.” Unfortunately, up to date, geographic information systems (GIS) miss the capability of geometric reasoning with extended objects. The aim of the paper is to present a mathematical apparatus for approximate geometric reasoning with extended objects that is usable in GIS. In the paper we discuss the fuzzy logic (Aliev and Tserkovny, 2011) as a reasoning system for geometry of extended objects, as well as a basis for fuzzification of the axioms of incidence geometry. The same fuzzy logic was used for fuzzification of Euclid's first postulate. Fuzzy equivalence relation “extended lines sameness” is introduced. For its approximation we also utilize a fuzzy conditional inference, which is based on proposed fuzzy “degree of indiscernibility” and “discernibility measure” of extended points.

## 1. Introduction

In [1–4] it was mentioned that there are numerous approaches by mathematicians to restore Euclidean Geometry from a different set of axioms, based on primitives that have extension in space. An approach, aimed at augmenting existent axiomatization of Euclidean geometry with grades of validity for axioms (fuzzification), is also presented in [1–4]. But in contrast with [1–4], where the* Lukasiewicz* logic was only proposed as the basis for “fuzzification” of axioms and no proofs were presented for both fuzzy predicates and fuzzy axiomatization of incidence geometry, we use fuzzy logic from [5] for all necessary mathematical purposes to fill up above-mentioned “gap.”

## 2. Axiomatic Geometry and Extended Objects

### 2.1. Geometric Primitives and Incidence

Similarly to [1–3, 6–8] we will use the following axioms from [9]. These axioms formalize the behaviour of points and lines in incident geometry, as it was defined in [1]:

(*I*1) *For every two distinct points p* and* q*,* at least one line l exists that is incident with p* and* q*.
(*I*2) *Such a line is unique*.
(*I*3) *Every line is incident with at least two points*.
(*I*4) *At least three points exist that are not incident with the same line*.

The uniqueness axiom (*I*2) ensures that geometrical constructions are possible. Geometric constructions are sequential applications of construction operators. An example of a construction operator is the following:

 
*Connect*:* point* ×* point* →* line*.

Take two points as an input and return the line through them. For* connect* to be a well-defined mathematical function, the resulting line needs always to exist and needs to be unique. Other examples of geometric construction operators of 2D incidence geometry are the following:

 
*Intersect*:* line* ×* line* →* point*.
 
*Parallel through point*:* line* ×* point* →* line.*

The axioms of incidence geometry form a proper subset of the axioms of Euclidean geometry. Incidence geometry allows for defining the notion of parallelism of two lines as a derived concept but does not permit expressing betweenness or congruency relations, which are assumed primitives in Hilbert's system [9]. The complete axiom set of Euclidean geometry provides a greater number of construction operators than incidence geometry. Incidence geometry has very limited expressive power when compared with the full axiom system.

The combined incidence axioms (*I*1) and (*I*2) state that it is always possible to connect two distinct points by a unique line. In case of coordinate points* a* and* b*, Cartesian geometry provides a formula for constructing this unique line:

(1) $$\begin{array}{c} l=\left\{\;a+t\left(\;b-a\;\right)\;|\;t\in R\;\right\}. \end{array}$$

As it was shown in [1–4], when we want to connect two extended geographic objects in a similar way, there is no canonical way of doing so. We cannot refer to an existing model like the Cartesian algebra. Instead, a new way of interpreting geometric primitives must be found, such that the interpretation of the incidence relation respects the uniqueness property (*I*2).

Similarly to [1–4] we will refer to extended objects that play the geometric role of points and lines by* extended points* and* extended lines*, respectively.  Section 3 gives a brief introduction on proposed fuzzy logic and discusses possible interpretations of fuzzy predicates for extended geometric primitives. The fuzzy logic from [5] is introduced as possible formalism for approximate geometric reasoning with extended objects and based on extended geometric primitives fuzzification of the incidence axioms (*I*1)*–*(*I*4) is investigated.

## 3. Fuzzification of Incidence Geometry

### 3.1. Proposed Fuzzy Logic

Let, ∀*p*, *q* ∈ [0,1] and continuous function (*p*, *q*) = *p* − *q*, which defines a distance between *p* and *q*. Notice that *F*(*p*, *q*) ∈ [−1,1], where *F*(*p*, *q*)^min^ = − 1 and *F*(*p*, *q*)^max^ = 1. When normalized, the value of *F*(*p*, *q*) is defined as follows:

(2) Fp,qnormFp,q−Fp,qminFp,qmax−Fp,qmin=Fp,q+12=p−q+12.

It is clear that  *F*(*p*, *q*)^norm^ ∈ [0,1]. This function represents the value of “closeness” between two values (potentially* antecedent* and* consequent*), defined within single interval, which therefore could play significant role in formulation of an implication operator in a fuzzy logic. Before proving that *I*(*p*, *q*) is defined as

(3) $$\begin{array}{c} I\left(\;p,q\;\right)=\left\{\begin{array}{cc} 1-F{\left(p,q\right)}^{norm}, & p>q;\\ 1, & p\leq q \end{array}\right. \end{array}$$

and *F*(*p*, *q*)^norm^ is from (2), let us show some basic operations in proposed fuzzy logic. Let us designate the truth values of logical* antecedent P* and* consequent Q* as *T*(*P*) = *p* and *T*(*Q*) = *q*, respectively. Then relevant set of proposed fuzzy logic operators is shown in Table 1. To get the truth values of these definitions we use well-known logical properties such as *p* → *q* = ¬*p*∨*q*; *p*∧*q* = ¬(¬*p*∨¬*q*) and the like.

In other words in [5] we proposed a new many-valued system, characterized by the set of base* union* (∪) and* intersection* (∩) operations with relevant* complement*, defined as *T*(¬*P*) = 1 − *T*(*P*). In addition, the operators ↓ and ↑ are expressed as negations of the ∪ and ∩ correspondingly. For this matter let us pose the problem very explicitly.

We are working in many-valued system, which for present purposes is all or some of the real interval *ℜ* = [0, 1]. As was mentioned in [5], the rationales there are more than ample for all practical purposes; the following set {0, 0.1, 0.2,…, 0.9, 1} of 11 values is quite sufficient, and we will use this set *V*_11_ in our illustration.  Table 2 shows the operation* implication* in proposed fuzzy logic.

### 3.2. Geometric Primitives as Fuzzy Predicates

It is well known that in Boolean predicate logic atomic statements are formalized by predicates. Predicates that are used in the theory of incidence geometry may be denoted by *p*(*a*) (“*a* is a point”), *l*(*a*) (“*a* is a line”), and inc⁡(*a*, *b*) (“*a* and* b* are incident”). The predicate expressing equality can be denoted by eq⁡(*a*, *b*) (“*a* and* b* are equal”). Traditionally predicates are interpreted by crisp relations. For example, eq: *N* × *N* → {0, 1} is a function that assigns 1 to every pair of equal objects and 0 to every pair of distinct objects from the set* N*. Of course, predicates like *p*(·) or *l*(·) which accept only one symbol as an input are* unary*, whereas* binary* predicates, like inc⁡(·, ·) and eq⁡(·, ·), accept pairs of symbols as an input. In a fuzzy predicate logic, predicates are interpreted by fuzzy relations, instead of crisp relations. For example, a binary fuzzy relation eq is a function eq: *N* × *N* → [0, 1], assigning a real number *λ* ∈ [0, 1] to* every* pair of objects from* N*. In other words, every two objects of *N* are equal to some degree. The degree of equality of two objects* a* and* b* may be 1 or 0 as in the crisp case but may as well be 0.9, expressing that* a* and* b* are* almost* equal. In [1–4] the fuzzification of *p*(·), *l*(·), inc⁡(·), and eq⁡(·) predicates was proposed.

Similarly to [1–4] we define a bounded subset Dom⊆*R* ^2^ as the domain for our geometric exercises. Predicates are defined for two-dimensional subsets *A*, *B*, *C*,…, of Dom and assume values in [0, 1]. We may assume two-dimensional subsets and ignore subsets of lower dimension, because every measurement and every digitization introduces a minimum amount of location uncertainty in the data [2]. For the point-predicate *p*(·) the result of Cartesian geometry involves a Cartesian point that does not change when the point is rotated: rotation-invariance seems to be a main characteristic of “point likeness” with respect to geometric operations; it should be kept when defining a fuzzy predicate expressing the “point likeness” of extended subsets of *R* ^2^. As a preliminary definition let

**Figure 1:**
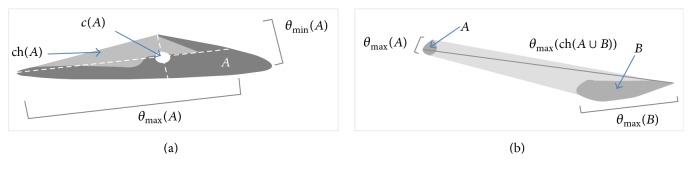
(a) Minimal and maximal diameter of a set* A* of Cartesian points. (b) Grade of distinctness *dc*(*A*, *B*) of* A* and* B*.

(4) θminA=mint⁡chA∩cA+t·Rα·0,1T ∣ t∈R,θmaxA=maxt⁡chA∩cA+t·Rα·0,1T ∣ t∈R

be the minimal and maximal diameter of the convex hull ch(*A*) of *A*⊆Dom, respectively. The convex hull regularizes the sets* A* and* B* and eliminates irregularities. *c*(*A*) denotes the centroid of ch(*A*), and *R*_*α*_ denotes the rotation matrix by angle *α* (Figure 1(a)) [1–4].

Since* A* is bounded, ch(*A*) and *c*(*A*) exist. We can now define the fuzzy point-predicate *p*(·) by

(5) $$\begin{array}{c} p\left(\;A\;\right)=\frac{\theta_{min}\left(A\right)}{\theta_{max}\left(A\right)}. \end{array}$$

*A*⊆Dom⁡*p*(·) expresses the degree to which the convex hull of a Cartesian point set* A* is rotation-invariant: if *p*(*A*) = 1, then ch(*A*) is perfectly rotation-invariant; it is a disc. Here, *θ*_max_(*A*) ≠ 0 always holds, because* A* is assumed to be two-dimensional. Converse to *p*(·), the fuzzy line-predicate

(6) $$\begin{array}{c} l\left(\;A\;\right)=1-p\left(\;A\;\right). \end{array}$$

Let us express the degree to which a Cartesian point set *A*⊆Dom is sensitive to rotation. Since we only regard convex hulls, *l*(·) disregards the detailed shape and structure of* A* but only measures the degree to which* A* is directed.

A fuzzy version of the incidence-predicate inc⁡(·, ·) is a binary fuzzy relation between Cartesian point sets, *B*⊆Dom:

(7) inc⁡A,B=max⁡chA∩chBchA,chA∩chBchB

measures the relative overlaps of the convex hulls of* A* and* B* and selects the greater one. Here |ch(*A*)| denotes the area occupied by ch(*A*). The greater inc⁡(*A*, *B*), “the more incident”* A* and* B*: if *A*⊆*B* or *B*⊆*A*, then inc⁡(*A*, *B*) = 1, and* A* and* B* are considered* incident to degree one*.

Conversely to inc⁡(·, ·), a graduated equality predicate eq⁡(·, ·) between the bounded Cartesian point sets *A*, *B*⊆Dom can be defined as follows:

(8) eq⁡A,B=min⁡chA∩chBchA,chA∩chBchB,

where eq⁡(*A*, *B*) measures the minimal relative overlap of* A* and* B*, whereas ¬eq⁡(*A*, *B*) = 1 − eq⁡(*A*, *B*) measures the degrees to which the two point sets do not overlap: if eq⁡(*A*, *B*) ≈ 0, then* A* and* B* are “almost disjoint.”

The following measure of “distinctness of points”, dp(·), of two extended objects tries to capture this fact (Figure 1(b)). We define

(9) $$\begin{array}{c} dp\left(\;A,B\;\right)=\max\left(\;0,1-\frac{\max\left(\theta_{max}\left(A\right),\theta_{max}\left(B\right)\right)}{\theta_{max}\left(ch\left(A\cup B\right)\right)}\;\right), \end{array}$$

where dp(*A*, *B*) expresses the degree to which ch(*A*) and ch(*B*) are distinct: the greater dp(*A*, *B*), the more* A* and* B* behave like distinct Cartesian points with respect to connection. Indeed, for Cartesian points* a* and* b*, we would have dp(*A*, *B*) = 1. If the distance between the Cartesian point sets* A* and* B* is infinitely big, then dp(*A*, *B*) = 1 as well. If max⁡(*θ*_max_(*A*), *θ*_max_(*B*)) > *θ*_max_(ch(*A* ∪ *B*)), then dp(*A*, *B*) = 0.

### 3.3. Formalization of Fuzzy Predicates

To formalize fuzzy predicates, defined in Section 3.2, both* implication *→ and* conjunction* operators are defined as in Table 1:

(10) A∧B=a+b2,a+b>1,0,a+b≤1,

(11) A⟶B=1−a+b2,a>b,1,a≤b.

In our further discussions we will also use the* disjunction* operator from the same table:

(12) $$\begin{array}{c} A\vee B=\left\{\begin{array}{cc} \frac{a+b}{2}, & a+b<1,\\ 1, & a+b\geq 1. \end{array}\right. \end{array}$$

Now let us redefine the set of fuzzy predicates (7)–(9), using proposed fuzzy logic's operators.

**Proposition 1 .**

If fuzzy predicate *inc*⁡(·, ·) is defined as in (7) and* conjunction* operator is defined as in (10), then

(13) $$\begin{array}{c} \mathrm{inc}\left(\;A,B\;\right)=\left\{\begin{array}{cc} \frac{a+b}{2a}, & a+b>1,\;\;a<b,\\ \frac{a+b}{2b}, & a+b>1,\;\;a>b,\\ 0, & a+b\leq 1. \end{array}\right. \end{array}$$

**Proof**

Let us present (7) as follows:

(14) $$\begin{array}{c} \mathrm{inc}\left(\;A,B\;\right)=\frac{\left|A\cap B\right|}{\min\left(\left|A\right|,\left|B\right|\right)}. \end{array}$$

And given that

(15) $$\begin{array}{c} \min\left(\;\left|\;A\;\right|,\left|\;B\;\right|\;\right)=\frac{a+b-\left|a-b\right|}{2}, \end{array}$$

from (7) and (10) we are getting (13).

It is important to notice that for the case when *a* + *b* > 1 in (13) the value of inc⁡(*A*, *B*) ≥ 1, which means that (13) in fact reduced into the following:

(16) inc⁡A,B=1,a+b>1,  a=b,  a>0.5,  b>0.5,0,a+b≤1.

**Proposition 2 .**

If fuzzy predicate *eq*⁡(·, ·) is defined as in (8) and* disjunction* operator is defined as in (12), then

(17) $$\begin{array}{c} eq\left(\;A,B\;\right)=\left\{\begin{array}{cc} \frac{a+b}{2b}, & a+b>1,\;\;a<b,\\ \frac{a+b}{2a}, & a+b>1,\;\;a>b,\\ 0, & a+b\leq 1. \end{array}\right. \end{array}$$

**Proof**

Let us rewrite (8) in the following way:

(18) $$\begin{array}{c} eq\left(\;A,B\;\right)=\min\left(\;\frac{A\cap B}{A},\frac{A\cap B}{B}\;\right). \end{array}$$

Let us define *P* = *A*∩*B*/*A* and *Q* = *A*∩*B*/*B*, and given (10) we have got the following:

(19) P=a+b2a,a+b>1,0,a+b≤1,Q=a+b2b,a+b>1,0,a+b≤1.

Therefore, given (15), we have the following.

Let us use (19) in the expression of min in (15) and first find the following:

(20) P+Qa+b2a+a+b2b,a+b>1,0,a+b≤1=a+b22ab,a+b>1,0,a+b≤1.

In the meantime we can show that the following is also taking place:

(21) P−Qa+b2a−a+b2b,a+b>1,0,a+b≤1=b2−a22ab,a+b>1,0,a+b≤1.

From (21) we are getting

(22) $$\begin{array}{c} \left|\;P-Q\;\right|=\left\{\begin{array}{cc} \frac{b^{2}-a^{2}}{2ab}, & a+b>1,\;\;b>a,\\ \frac{a^{2}-b^{2}}{2ab}, & a+b>1,\;\;a>b,\\ 0, & a+b\leq 1. \end{array}\right. \end{array}$$

But from (18) we have the following:

(23) eqA,Bmin⁡P,Q=P+Q−P−Q2=a+b2−b2+a22ab,a+b>1,  b>a,a+b2−a2+b24ab,a+b>1,  a>b,0,a+b≤1=a+b2b,a+b>1,  a<b,a+b2a,a+b>1,  a>b,0,a+b≤1.

**Corollary 3 .**

If fuzzy predicate *eq*⁡(*A*, *B*) is defined as (23), then the following type of* transitivity* is taking place:

(24) $$\begin{array}{c} eq\left(\;A,C\;\right)⟶eq\left(\;A,B\;\right)\wedge eq\left(\;B,C\;\right), \end{array}$$

where *A*, *B*, *C*⊆Dom and Dom is* partially ordered* space that is either *A*⊆*B*⊆*C* or vice versa (note: both* conjunction* and* implication* operations are defined in Table 1).

**Proof**

From (17) we have

(25) eq⁡A,B=a+b2b,a+b>1,  a<b,a+b2a,a+b>1,  a>b,0,a+b≤1,eq⁡B,C=b+c2c,b+c>1,  b<c,b+c2b,b+c>1,  b>c,0,b+c≤1;

then

(26) eq⁡A,B∧eq⁡B,C=eq⁡A,B+eq⁡B,C2,eq⁡A,B+eq⁡B,C>1,0,eq⁡A,B+eq⁡B,C≤1.

Meanwhile, from (17) we have the following:

(27) $$\begin{array}{c} \mathrm{eq}\left(\;A,C\;\right)=\left\{\begin{array}{cc} \frac{a+c}{2c}, & a+c>1,\;\;a<c,\\ \frac{a+c}{2a}, & a+c>1,\;\;a>c,\\ 0, & a+c\leq 1. \end{array}\right. \end{array}$$

*Case  1 (a* < *b* < *c).* From (26) we have

(28) eq⁡A,B∧eq⁡B,C2=a+b2b+b+c2c=ac+2bc+b24bc.

From (27) and (28) we have to prove that

(29) $$\begin{array}{c} \frac{a+c}{2c}⟶\frac{ac+2bc+b^{2}}{4bc}. \end{array}$$

But (29) is the same as (2*ab* + 2*bc*)/4*bc* → (*ac* + 2*bc* + *b* ^2^)/4*bc*, from which we get 2*ab* → *ac* + *b* ^2^.

From definition of* implication* in fuzzy logic (11) and since for *a* < *b* < *c* condition 2*ab* < *ac* + *b* ^2^ is taking place, therefore 2*ab* → *ac* + *b* ^2^ = 1.

*Case  2 (a* > *b* > *c).* From (26) we have

(30) eq⁡A,B∧eq⁡B,C2=a+b2a+b+c2b=ac+2ab+b24ab.

From (27) and (30) we have to prove that

(31) $$\begin{array}{c} \frac{a+c}{2a}⟶\frac{ac+2ab+b^{2}}{4ab}. \end{array}$$

But (31) is the same as (2*ab* + 2*bc*)/4*ab* → (*ac* + 2*ab* + *b* ^2^)/4*ab*, from which we get 2*bc* → *ac* + *b* ^2^.

From definition of* implication* in fuzzy logic (11) and since for *a* > *b* > *c* condition 2*bc* < *ac* + *b* ^2^ is taking place, therefore 2*bc* → *ac* + *b* ^2^ = 1.

**Proposition 4 .**

If fuzzy predicate *dp*(·, ·) is defined as in (9) and* disjunction* operator is defined as in (12), then

(32) $$\begin{array}{c} dp\left(\;A,B\;\right)=\left\{\begin{array}{cc} 1-a, & a+b\geq 1,\;\;a\geq b,\\ 1-b, & a+b\geq 1,\;\;a<b,\\ 0, & a+b<1. \end{array}\right. \end{array}$$

**Proof**

From (9) we get the following:

(33) $$\begin{array}{c} dp\left(\;A,B\;\right)=\max\left\{\;0,1-\frac{\max\left(A,B\right)}{A\cup B}\;\right\}. \end{array}$$

Given that max⁡(*A*, *B*) = (*a* + *b* + |*a* − *b*|)/2, from (33) and (9) we are getting the following:

(34) dpA,B=max⁡0,1−a+b+a−ba+b,a+b<1,max⁡0,1−a+b+a−b2,a+b≥1.

(1) From (34) we have
  (35) max⁡0,1−a+b+a−b2=max⁡0,2−a−b−a−b2=1−a,a+b≥1,  a≥b,1−b,a+b≥1,  a<b.
(2) Also from (34) we have
  (36) max⁡0,1−a+b+a−ba+b=max⁡0,−a−ba+b=0,a+b<1.

From both (35) and (36) we have gotten that

(37) $$\begin{array}{c} dp\left(\;A,B\;\right)=\left\{\begin{array}{cc} 1-a, & a+b\geq 1,\;\;a\geq b,\\ 1-b, & a+b\geq 1,\;\;a<b,\\ 0, & a+b<1. \end{array}\right. \end{array}$$

### 3.4. Fuzzy Axiomatization of Incidence Geometry

Using the fuzzy predicates formalized in Section 3.3, we propose the set of axioms as fuzzy version of incidence geometry in the language of a fuzzy logic [5] as follows:

$\left(I1^{\prime }\right)$ *⁡*dp(*a*, *b*) → sup_*c*_⁡[*l*(*c*)∧inc⁡(*a*, *c*)∧inc⁡(*b*, *c*)];
$\left(I2^{\prime }\right)$ *⁡*dp(*a*, *b*) → [*l*(*c*) → [inc⁡(*a*, *c*) → [inc⁡(*b*, *c*) → *l*(*c*′) → [inc⁡(*a*, *c*′) → [inc⁡(*b*, *c*′) → eq⁡(*c*, *c*′)]]]]];
$\left(I3^{\prime }\right)$ *⁡l*(*c*) → sup_*a*,*b*_⁡{*p*(*a*)∧*p*(*b*)∧¬eq⁡(*a*, *b*)∧inc⁡(*a*, *c*)∧inc⁡(*b*, *c*)};
$\left(I4^{\prime }\right)$ *⁡*sup_*a*,*b*,*c*,*d*_⁡[*p*(*a*)∧*p*(*b*)∧*p*(*c*)∧*l*(*d*) → ¬(inc⁡(*a*, *d*)∧inc⁡(*b*, *d*)∧inc⁡(*c*, *d*))].

In axioms (*I*1′)–(*I*4′) we also use a set of operations (10)–(12).

**Proposition 5 .**

If fuzzy predicates *dp*⁡(·, ·) and *inc*⁡(·, ·) are defined like (37) and (13), respectively, then axiom (*I*1′) is fulfilled for the set of logical operators from a fuzzy logic [5]. (*For every two distinct points a and b, at least one line l exists, i.e., incident with a and b.*)

**Proof**

From (16)

(38) inc⁡A,C=1,a+c>1,  a=c,  a>0.5,  c>0.5,0,a+c≤1,inc⁡B,C=1,b+c>1,  b=c,  b>0.5,  c>0.5,0,b+c≤1,inc⁡A,C∧inc⁡B,C=inc⁡A,C+inc⁡B,C2≡1,

sup_*c*_⁡[*l*(*c*)∧inc⁡(*a*, *c*)∧inc⁡(*b*, *c*)] and given (38) sup_*c*_⁡[*l*(*c*)∧1] = 0∧1 ≡ 0.5. From (37) and (10) dp(*a*, *b*) ≤ 0.5 and we are getting dp(*a*, *b*) ≤ sup_*c*_⁡[*l*(*c*)∧inc⁡(*a*, *c*)∧inc⁡(*b*, *c*)].

**Proposition 6 .**

If fuzzy predicates *dp*(·, ·), *eq*(·, ·), and *inc*⁡(·, ·) are defined like (37), (17), and (16), respectively, then axiom (*I*2′) is fulfilled for the set of logical operators from a fuzzy logic [5] (for every two distinct points* a* and* b*, at least one line* l* exists, i.e., incident with a and b, and such a line is unique).

**Proof**

Let us take a look at the following implication:

(39) $$\begin{array}{c} inc\left(\;b,c^{\prime }\;\right)⟶eq\left(\;c,c^{\prime }\;\right). \end{array}$$

But from (27) we have

(40) $$\begin{array}{c} eq\left(\;C,C^{\prime }\;\right)=\left\{\begin{array}{cc} \frac{c+c^{\prime }}{2c^{\prime }}, & c+c^{\prime }>1,\;\;c<c^{\prime },\\ \frac{c+c^{\prime }}{2c}, & c+c^{\prime }>1,\;\;c>c^{\prime },\\ 0, & c+c^{\prime }\leq 1. \end{array}\right. \end{array}$$

And from (16)

(41) $$\begin{array}{c} inc\left(\;B,C\;\right)=\left\{\begin{array}{cc} 1, & b+c>1,\;\;b=c,\;\;b>0.5,\;\;c>0.5,\\ 0, & b+c\leq 1. \end{array}\right. \end{array}$$

From (40) and (41) we see that inc⁡(*B*, *C*) ≤ eq⁡(*C*, *C*′), which means that

(42) $$\begin{array}{c} inc\left(\;b,c^{\prime }\;\right)⟶\mathrm{eq}\left(\;c,c^{\prime }\;\right)\equiv 1; \end{array}$$

therefore the following is also true:

(43) $$\begin{array}{c} \mathrm{inc}\left(\;a,c^{\prime }\;\right)⟶\left[\;inc\left(\;b,c^{\prime }\;\right)⟶eq\left(\;c,c^{\prime }\;\right)\;\right]\equiv 1. \end{array}$$

Now let us take a look at the following implication: inc(*b*, *c*) → *l*(*c*′). Since inc(*b*, *c*) ≥ *l*(*c*′), we are getting inc(*b*, *c*) → *l*(*c*′) ≡ 0. Taking into account (43) we have the following:

(44) $$\begin{array}{c} \begin{array}{c} inc\left(b,c\right)⟶l\left(c^{\prime }\right)⟶\left[inc\left(a,c^{\prime }\right)⟶\left[inc\left(b,c^{\prime }\right)⟶eq\left(c,c^{\prime }\right)\right]\right]\equiv 1. \end{array} \end{array}$$

Since, from (16), inc(*a*, *c*) ≤ 1, then with taking into account (44) we have gotten the following: 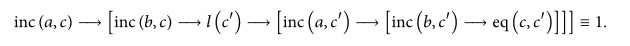
(45)Since *l*(*c*) ≤ 1, from (45) we are getting

(46) $$\begin{array}{c} \begin{array}{c} l\left(c\right)⟶\left[inc\left(a,c\right)⟶\left[inc\left(b,c\right)⟶l\left(c^{\prime }\right)⟶\left[inc\left(a,c^{\prime }\right)⟶\left[inc\left(b,c^{\prime }\right)⟶eq\left(c,c^{\prime }\right)\right]\right]\right]\right] \end{array}\equiv 1. \end{array}$$

Finally, because dp(*a*, *b*) ≤ 1 we have
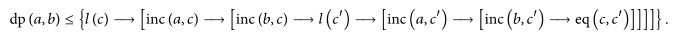
(47)

**Proposition 7 .**

If fuzzy predicates *eq*(·, ·) and *inc*⁡(·, ·) are defined like (17) and (16), respectively, then axiom (*I*3′) is fulfilled for the set of logical operators from a fuzzy logic [5]. (*Every line is incident with at least two points.*)

**Proof**

It was already shown in (38) that

(48) $$\begin{array}{c} inc\left(\;a,c\;\right)\wedge inc\left(\;b,c\;\right)=\frac{inc\left(a,c\right)+inc\left(b,c\right)}{2}\equiv 1. \end{array}$$

And from (17) we have

(49) $$\begin{array}{c} eq\left(\;A,B\;\right)=\left\{\begin{array}{cc} \frac{a+b}{2b}, & a+b>1,\;\;a<b,\\ \frac{a+b}{2a}, & a+b>1,\;\;a>b,\\ 0, & a+b\leq 1. \end{array}\right. \end{array}$$

The negation ¬eq(*A*, *B*) will be

(50) $$\begin{array}{c} \neg eq\left(\;A,B\;\right)=\left\{\begin{array}{cc} \frac{b-a}{2b}, & a+b>1,\;\;a<b,\\ \frac{a-b}{2a}, & a+b>1,\;\;a>b,\\ 1, & a+b\leq 1. \end{array}\right. \end{array}$$

Given (38) and (50) we get

(51) ¬eqA,B∧1=1+b−a/2b2,a+b>1,  a<b,1+a−b/2a2,a+b>1,  a>b,1,a+b≤1=3b−a4b,a+b>1,  a<b,3a−b4a,a+b>1,  a>b,1,a+b≤1,

since ¬eq(*A*, *B*)∧1 ≡ 0.5∣*a* = 1, *b* = 1, from which we are getting sup_*a*,*b*_⁡{*p*(*a*)∧*p*(*b*)∧¬eq(*a*, *b*)∧inc(*a*, *c*)∧inc(*b*, *c*)} = 1∧0.5 = 0.75.

And given that *l*(*c*) ≤ 0.75 we are getting *l*(*c*) → sup_*a*,*b*_⁡{*p*(*a*)∧*p*(*b*)∧¬eq(*a*, *b*)∧inc(*a*, *c*)∧inc(*b*, *c*)} ≡ 1.

**Proposition 8 .**

If fuzzy predicate *inc*(·, ·) is defined like (16), then axiom (*I*4′) is fulfilled for the set of* logical operators* from a fuzzy logic [5]. (*At least three points exist that are not incident with the same line.*)

**Proof**

From (16) we have

(52) incA,D=1,a+d>1,  a=d,  a>0.5,  d>0.5,0,a+d≤1,incB,D=1,b+d>1,  b=d,  b>0.5,  d>0.5,0,b+d≤1,incC,D=1,c+d>1,  c=d,  c>0.5,  d>0.5,0,c+d≤1.

But from (38) we have

(53) $$\begin{array}{c} inc\left(\;A,D\;\right)\wedge inc\left(\;B,D\;\right)=\frac{inc\left(A,D\right)+inc\left(B,D\right)}{2}\equiv 1 \end{array}$$

and (inc(*a*, *d*)∧inc(*b*, *d*)∧inc(*c*, *d*)) = 1∧inc(*c*, *d*) ≡ 1, where we have  ¬(inc(*a*, *d*)∧inc(*b*, *d*)∧inc(*c*, *d*)) ≡ 0. Since *l*(*d*) ≡ 0∣*d* = 1 we are getting *l*(*d*) = ¬(inc(*a*, *d*)∧inc(*b*, *d*)∧inc(*c*, *d*)), which could be interpreted like  *l*(*d*)→¬(inc(*a*, *d*)∧inc(*b*, *d*)∧inc(*c*, *d*)) = 1, from which we finally get sup_*a*,*b*,*c*,*d*_⁡[*p*(*a*)∧*p*(*b*)∧*p*(*c*)∧1] ≡ 1.

### 3.5. Equality of Extended Lines Is Graduated

**Figure 2:**
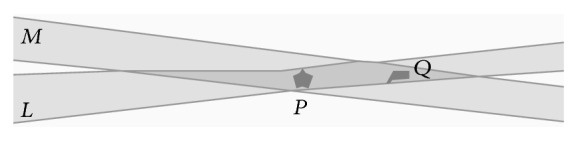
Two extended points do not uniquely determine the location of an incident extended line.

In [10] it was shown that the location of the extended points creates a constraint on the location of an incident extended line. It was also mentioned that in traditional geometry this location constraint fixes the position of the line uniquely. It is not true in case of extended points and lines. Consequently Euclid's first postulate does not apply: Figure 2 shows that if two distinct extended points* P* and* Q* are incident (i.e., overlap) with two extended lines* L* and* M*, then* L* and* M* are not necessarily equal.

Yet, in most cases,* L* and* M* are “*closer together,*” that is, “*more equal*” than arbitrary extended lines that have only one or no extended point in common. The further* P* and* Q* move apart from each other, the more similar* L* and* M* become. One way to model this fact is to allow* degrees of equality* for extended lines. In other words, the equality relation* is* graduated: it allows for not only Boolean values, but also values in the whole interval [0, 1].

### 3.6. Incidence of Extended Points and Lines

As it was demonstrated in [10], there is a reasonable assumption to classify an extended point and an extended line as incident, if their extended representations in the underlying metric space overlap. We do this by modelling incidence by the subset relation.

**Definition 9 .**

For an extended point* P* and an extended line* L* we define the* incidence* relation by

(54) $$\begin{array}{c} R_{inc}\left(\;P,L\;\right)≔\left(\;P\subseteq L\;\right)\in \left\{\;0,1\;\right\}, \end{array}$$

where the subset relation ⊆ refers to* P* and* L* as subsets of the underlying metric space.

The extended incidence relation (54) is a Boolean relation, assuming either the truth value 1 (*true*) or the truth value 0 (*false*). It is well known that since a Boolean relation is a special case of a graduated relation, that is, since {0, 1} ⊂ [0, 1], we will be able to use relation (54) as part of fuzzified Euclid's first postulate later on.

### 3.7. Equality of Extended Points and Lines

As stated in previous sections, equality of extended points and equality of extended lines are a matter of degree. Geometric reasoning with extended points and extended lines relies heavily on the metric structure of the underlying coordinate space. Consequently, it is reasonable to model graduated equality as inverse to distance.

#### 3.7.1. Metric Distance

In [10] it was mentioned that a pseudo metric distance, or pseudo metric, is a map *d* : *M* ^2^ → *ℜ* ^+^ from domain* M* into the positive real numbers (including zero), which is minimal and symmetric and satisfies the triangle inequality:

(55) ∀p,q∈0,1⇓dp,p=0dp,q=dq,pdp,q+dq,r≥dp,r.

*d* is called a metric, if the following takes place:

(56) dp,q=0⟺p=q.

Well-known examples of metric distances are the Euclidean distance or the Manhattan distance. The “upside-down-version” of a pseudo metric distance is a fuzzy equivalence relation with respect to a proposed* t*-norm. The next section introduces the logical connectives in a proposed* t*-norm fuzzy logic. We will use this particular fuzzy logic to formalize Euclid's first postulate for extended primitives in Section 5. The reason for choosing a proposed fuzzy logic is its strong connection to metric distance.

#### 3.7.2. The* t*-Norm

**Proposition 10 .**

In proposed fuzzy logic the operation of conjunction (10) is a* t*-norm.

**Proof**

The function *f*(*p*, *q*) is a* t*-norm if the following hold:

(1) *Commutativity*: *p*∧*q* = *q*∧*p*.
(2) *Associativity*: (*p*∧*q*)∧*r* = *p*∧(*q*∧*r*).
(3) *Monotonicity*: *p* ≤ *q*, *p*∧*r* ≤ *q*∧*r*.
(4) *Neutrality*: 1∧*p* = *p*.
(5) *Absorption*: 0∧*p* = 0.

*Commutativity*. Consider

(57) fp,q=P∩Q=p+q2,p+q>1,0,p+q≤1,fq,p=Q∩P=q+p2,q+p>1,0,q+p≤1;

therefore *f*(*p*, *q*) = *f*(*q*, *p*).

*Associativity*

*Case  1 (f*(*p*, *q*)∧*r).* Consider

(58) fp,qp+q2,p+q>1⟹fp,q∧rfp,q+r2,fp,q+r>1,0,fp,q+r≤1=p+q+2r4,p+q2+r>1,0,p+q2+r≤1,

from where we have that

(59) $$\begin{array}{c} f_{1}\left(\;p,r\;\right)=\left\{\begin{array}{cc} \frac{p+q+2r}{4}, & p+q+2r>2,\\ 0, & p+q+2r\leq 2. \end{array}\right. \end{array}$$

In other words *f*_1_(*p*, *r*)⊆(0.5; 1]∣*p* + *q* + 2*r* > 2 and *f*_1_(*p*, *r*) = 0∣*p* + *q* + 2*r* ≤ 2.

For the case *p*∧*f*(*q*, *r*) we are getting results similar to (59):

(60) $$\begin{array}{c} f_{2}\left(\;p,r\;\right)=\left\{\begin{array}{cc} \frac{q+r+2p}{4}, & q+r+2p>2,\\ 0, & q+r+2p\leq 2, \end{array}\right. \end{array}$$

that is, *f*_2_(*p*, *r*)⊆(0.5; 1]∣*q* + *r* + 2*p* > 2 and *f*_2_(*p*, *r*) = 0∣*q* + *r* + 2*p* ≤ 2. Consider *f*_1_(*p*, *r*) ≈ *f*_2_(*p*, *r*).

*Monotonicity. *If *p* ≤ *q*⇒*p*∧*r* ≤ *q*∧*r*, then, given

(61) p∧r=p+r2,p+r>1,0,p+r≤1,q∧r=q+r2,q+r>1,0,q+r≤1,

we are getting (*p* + *r*)/2 ≤ (*q* + *r*)/2⇒*p* + *r* ≤ *q* + *r*⇒*p* ≤ *q*∣*p* + *r* > 1 and *q* + *r* > 1, whereas, for the case *p* + *r* ≤ 1, *q* + *r* ≤ 1⇒0 ≡ 0.

*Neutrality*. Consider

(62) 1∧p1+p2,1+p>1,0,1+p≤1=1+p2,p>0,0,p≤0=1+p2,p>0,0,p=0,

from which the following is apparent:

(63) $$\begin{array}{c} 1\wedge p=\left\{\begin{array}{cc} \frac{1+p}{2}, & p\in \left(0,1\right),\\ p, & p=0,\;\;p=1. \end{array}\right. \end{array}$$

*Absorption*. Consider

(64) $$\begin{array}{c} 0\wedge p=\left\{\begin{array}{cc} \frac{p}{2}, & p>1,\\ 0, & p\leq 1, \end{array}\right. \end{array}$$

since *p* ∈ [0,1]⇒0∧*p* ≡ 0.

#### 3.7.3. Fuzzy Equivalence Relations

As mentioned above, the “upside-down-version” of a pseudo metric distance is a fuzzy equivalence relation with respect to the proposed* t*-norm ∧. A fuzzy equivalence relation is a fuzzy relation *e* : *M* ^2^ → [0,1] on a domain* M*, which is reflexive, symmetric, and ∧-transitive:

(65) ∀p,q∈0,1⇓ep,p=1ep,q=eq,pep,q∧eq,r≤ep,r.

**Proposition 11 .**

If fuzzy equivalence relation is defined (Table 1) as

(66) ep,q=P⟷Q=1−p+q2,p>q,1,p=q,1−q+p2,p<q,

then conditions (65) are satisfied.

**Proof**

 

(*1) Reflexivity*. *e*(*p*, *p*) = 1 comes from (66) because *p* ≡ *p*.

(*2) Symmetricity*. *e*(*p*, *q*) = *e*(*q*, *p*). Consider the following:

(67) $$\begin{array}{c} e\left(\;p,q\;\right)=\left\{\begin{array}{cc} \frac{1-p+q}{2}, & p>q,\\ 1, & p=q,\\ \frac{1-q+p}{2}, & p<q, \end{array}\right. \end{array}$$

but

(68) $$\begin{array}{c} e\left(\;q,p\;\right)=\left\{\begin{array}{cc} \frac{1-q+p}{2}, & q>p,\\ 1, & q=p,\\ \frac{1-p+q}{2}, & q<p; \end{array}\right. \end{array}$$

therefore *e*(*p*, *q*) ≡ *e*(*q*, *p*).

(*3) Transitivity*. *e*(*p*, *q*)∧*e*(*q*, *r*) ≤ *e*(*p*, *r*)∣∀ *p*, *q*, *r* ∈ *L*[0,1]-lattice.

From (66) let

(69) F1p,r=ep,r=1−p+r2,p>r,1,p=r,1−r+p2,p<r,

(70) eq,r=1−q+r2,q>r,1,q=r,1−r+q2,q<r;

then

(71) F2p,r=ep,q∧eq,r=ep,q+eq,r2,ep,q+eq,r>1,0,ep,q+eq,r≤1.

But

(72) F2p,r=ep,q+eq,r2=1−p+q/2+1−q+r/22,p>q>r,1,p=q=r,1−q+p/2+1−r+q/22,p<q<r=2−p+r4,p>q>r,1,p=q=r,2−r+p4,p<r<r.

Now compare (72) and (69). It is apparent that *r* > *p*⇒(2 − *p* + *r*)/4 < (1 − *p* + *r*)/2⇔*r* − *p* < 2(*r* − *p*). The same is true for *p* > *r*⇒(2 − *r* + *p*)/4 < (1 − *r* + *p*)/2⇔*p* − *r* < 2(*p* − *r*). And lastly (*e*(*p*, *q*) + *e*(*q*, *r*))/2 ≡ *e*(*p*, *r*) ≡ 1, when *p* = *r*. Given that *F*_2_(*p*, *r*) = *e*(*p*, *q*)∧*e*(*q*, *r*) ≡ 0, *e*(*p*, *q*) + *e*(*q*, *r*) ≤ 1, we are getting the proof of the fact that *F*_2_(*p*, *r*) ≤ *F*_1_(*p*, *r*)⇔*e*(*p*, *q*)∧*e*(*q*, *r*) ≤ *e*(*p*, *r*)∣∀ *p*, *q*, *r* ∈ *L*[0,1].

Note that relation *e*(*p*, *q*) is called a* fuzzy equality relation*, if additionally separability holds: *e*(*p*, *q*) = 1⇔*p* = *q*. Let us define a* pseudo metric distance d*(*p*, *q*) for domain* M*, normalized to 1, as

(73) $$\begin{array}{c} e\left(\;p,q\;\right)=1-d\left(\;p,q\;\right). \end{array}$$

From (66) we are getting

(74) dp,q1+p−q2,p>q,0,p=q,1+q−p2,p<q=1+p−q2,p≠q,0,p=q.

#### 3.7.4. Approximate Fuzzy Equivalence Relations

**Figure 3:**
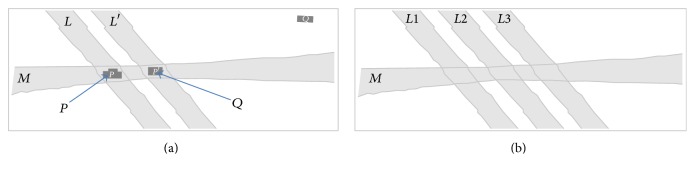
(a) Graduated equality of extended lines compels graduated equality of extended points. (b) Equality of extended lines is not transitive.

In [11] it was mentioned that* graduated equality* of* extended lines* compels* graduated equality* of* extended points*.  Figure 3(a) sketches a situation where two extended lines* L* and* M* intersect in an extended point* P*. If a third extended line *L*′ is very similar to* L*, its intersection with* M* yields an extended point *P*′ which is very similar to* P*. It is desirable to model this fact. To do so, it is necessary to allow graduated equality of extended points.

Figure 3(b) illustrates that an equality relation between extended objects need not be transitive. This phenomenon is commonly referred to as the Poincare paradox. The Poincare paradox is named after the famous French mathematician and theoretical physicist Poincare, who repeatedly pointed this fact out, for example, in [12], referring to indiscernibility in sensations and measurements. Note that this phenomenon is usually insignificant, if positional uncertainty is caused by stochastic variability. In measurements, the stochastic variability caused by measurement inaccuracy is usually much greater than the indiscernibility caused by limited resolution. For extended objects, this relation is reversed: the extension of an object can be interpreted as indiscernibility of its contributing points. In the present paper we assume that the extension of an object is being compared with the indeterminacy of its boundary. Gerla shows that modelling the Poincare paradox in* graduated context transitivity* may be replaced by a weaker form [13]:

(75) $$\begin{array}{c} e\left(\;p,q\;\right)\wedge e\left(\;q,r\;\right)\wedge \text{dis}\left(\;q\;\right)\leq e\left(\;p,r\;\right). \end{array}$$

Here dis : *M* → [0,1] is a lower-bound measure (*discernibility measure*) for the degree of transitivity that is permitted by* q.* A pair (*e*, dis) that is reflexive, symmetric, and weakly transitive (75) is called an* approximate fuzzy *∧*-equivalence relation*. Let us rewrite (75) as follows:

(76) $$\begin{array}{c} F_{2}\left(\;p,r\;\right)\wedge \text{dis}\left(\;q\;\right)\leq F_{1}\left(\;p,r\;\right), \end{array}$$

where *F*_2_(*p*, *r*), *F*_1_(*p*, *r*) are defined in (72) and (69) correspondingly. But

(77) ∀p,q,r ∣ p<q<r⟹F2p,r∧disq=2−r+p/4+disq2,2−r+p4+disq>1,0,2−r+p4+disq≤1,∀p,q,r ∣ p>q>r⟹F2p,r∧disq=2−p+r/4+disq2,2−p+r4+disq>1,0,2−p+r4+disq≤1.

From (77) in order to satisfy condition (76) we have

(78) ∀p,q,r ∣ p<q<r⟹disq>1−2−r+p4;∀p,q,r ∣ p>q>r⟹disq>1−2−p+r4

that is, we have

(79) $$\begin{array}{c} \text{dis}\left(\;q\;\right)≅\left\{\begin{array}{cc} \frac{2+\left|p-r\right|}{4}, & r\neq p,\\ 0, & r=p. \end{array}\right. \end{array}$$

By using (79) in (77) we are getting that ∀*p*, *q*, *r* ∈ [0,1]⇒*F*_2_(*p*, *r*)∧dis(*q*) ≡ 0.5. From (69) we are getting ∀*p*, *r* ∈ [0,1]⇒*F*_1_(*p*, *r*)∈[0.5,1] and subsequently inequality (76) holds.

In [11] it was also mentioned that an* approximate fuzzy *∧*-equivalence relation* is the upside-down-version of a so-called* pointless pseudo metric space *(*δ*, *s*):

(80) δp,p=0,δp,q=δq,p,δp,q∨δq,r∨sq≥δp,r.

Here, *δ* : *M* → *ℜ* ^+^ is a (not necessarily metric) distance between extended regions and *s* : *M* → *ℜ* ^+^ is a* size measure* and we are using an* operation disjunction* (12) also shown in Table 1. Inequality *δ*(*q*, *r*)∨*s*(*q*) ≥ *δ*(*p*, *r*) is a weak form of the triangle inequality. It corresponds to the weak transitivity (75) of the* approximate fuzzy *∧*-equivalence relation e*. In case the size of the domain* M* is normalized to 1,* e* and dis can be represented by [13]

(81) ep,q=1−δp,q,disq=1−sq.

**Proposition 12 .**

If a distance between extended regions *δ*(*p*, *q*) from (80) and* pseudo metric distance d*(*p*, *q*) for domain* M*, normalized to 1, are the same, that is, *δ*(*p*, *q*) = *d*(*p*, *q*), then inequality *δ*(*p*, *q*)∨*δ*(*q*, *r*)∨*s*(*q*) ≥ *δ*(*p*, *r*) holds.

**Proof**

From (74) we have

(82) δp,q=1+p−q2,p>q,0,p=q,1+q−p2,p<q,δq,r=1+q−r2,q>r,0,q=r,1+r−q2,q<r.

Given (82),

(83) δp,q∨δq,r1+p−q/2+1+q−r/22,δp,q+δq,r<1,  p>q>r,1,δp,q+δq,r≥1,0,p=q=r,1+q−p/2+1+r−q/22,δp,q+δq,r<1,  p<q<r=2+p−r4,δp,q+δq,r<1,  p>q>r,1,δp,q+δq,r≥1,0,p=q=r,2+r−p4,δp,q+δq,r<1,  p<q<r=2+p−r4,δp,q+δq,r<1,  p≠q≠r,1,δp,q+δq,r≥1,0,p=q=r,

but

(84) $$\begin{array}{c} \delta \left(\;p,r\;\right)=\left\{\begin{array}{cc} \frac{1+p-r}{2}, & p>r,\\ 0, & p=r,\\ \frac{1+r-p}{2}, & p<r. \end{array}\right. \end{array}$$

From (83) and (84) the following is apparent:

(85) $$\begin{array}{c} \delta \left(\;p,q\;\right)\vee \delta \left(\;q,r\;\right)\leq \delta \left(\;p,r\;\right). \end{array}$$

Now we have to show that size measure *s*(*q*) > 0. From (79) we have

(86) $$\begin{array}{c} s\left(\;q\;\right)=1-\text{dis}\left(\;q\;\right)=\left\{\begin{array}{cc} \frac{2-\left|p-r\right|}{4}, & r\neq p,\\ 1, & r=p. \end{array}\right. \end{array}$$

It is apparent that *s*(*q*)∈(0.25,1]∣∀ *r*, *p*, *q* ∈ [0,1]; therefore from (84), (85), and (86) *δ*(*p*, *q*)∨*δ*(*q*, *r*)∨*s*(*q*) ≥ *δ*(*p*, *r*) holds.

Noting *δ*(*p*, *r*) from (84) we have ∀*r*, *p* ∈ [0,1]⇒*δ*(*p*, *r*) = (1 + |*p* − *r*|)/2 ∈ [0,1]. But as it was mentioned in [10], given a* pointless pseudo metric space *(*δ*, *s*) for extended regions on a normalized domain, (81) define an* approximate fuzzy *∧*-equivalence relation *(*e*, dis) by simple logical negation. The so-defined equivalence relation on the one hand complies with the Poincare paradox, and on the other hand it retains enough information to link two extended points (or lines) via a third. For used fuzzy logic an example of a* pointless pseudo metric space* is the set of extended points with the following measures:

(87) δP,Q≔infdp,q ∣ p∈P,q∈Q,

(88) sP≔sup⁡dp,q ∣ p,q∈P.

It is easy to show that (86) and (87) are satisfied, because from (74) *d*(*p*, *q*)∈[0,1]∣∀ *r*, *p*, *q* ∈ [0,1]. A* pointless metric distance *of extended lines can be defined in the dual space [10]:

(89) δL,M≔inf⁡dl′,m′ ∣ l∈L,  m∈M,

(90) sL≔sup⁡dl′,m′ ∣ l,m∈L.

#### 3.7.5. Boundary Conditions for Granularity

**Figure 4:**
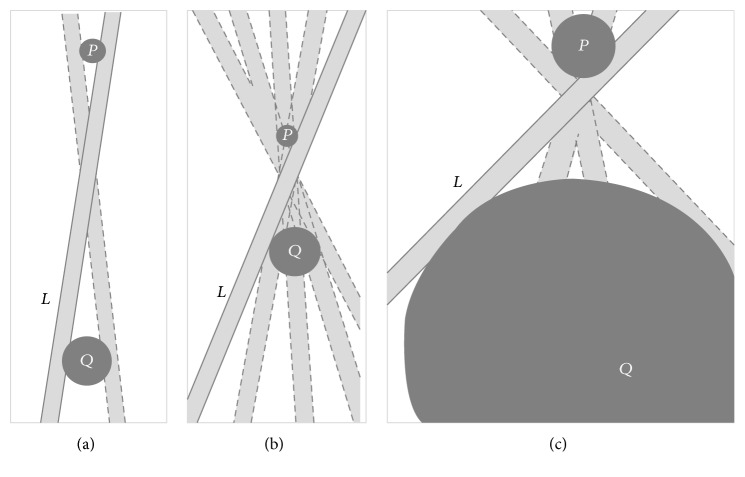
Size and distance matter.

As it was mentioned in [10], in exact coordinate geometry, points and lines do not have size. As a consequence, distance of points does not matter in the formulation of Euclid's first postulate. If points and lines are allowed to have extension, both size and distance matter.  Figure 4 depicts the location constraint on an extended line* L* that is incident with the extended points* P* and* Q*.

The location constraint can be interpreted as* tolerance in the position of L*. In Figure 4(a) the distance of* P* and* Q* is* large* with respect to the sizes of* P* and* Q* and with respect to the width of* L*. The resulting positional tolerance for* L* is* small*. In Figure 4(b), the distance of* P* and* Q* is* smaller* than that in Figure 4(a). As a consequence the positional tolerance for* L* becomes* larger*. In Figure 4(c),* P* and* Q* have the same distance as in Figure 4(a), but their sizes are increased. Again, positional tolerance of* L* increases. As a consequence, formalization of Euclid's first postulate for extended primitives must take all three parameters into account: the distance of the extended points, their size, and the size of the incident line.

**Figure 5:**
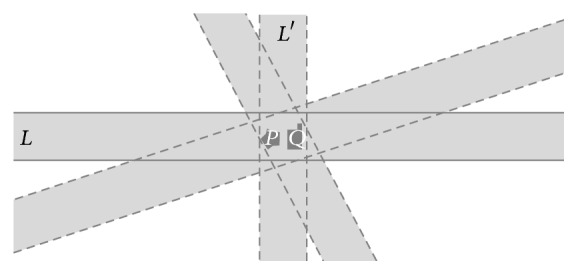
*P* and* Q* are indiscernible for* L*.

Figure 5 illustrates this case: despite the fact that* P* and* Q* are distinct extended points that are both incident with* L*, they do not specify any* directional constraint* for* L*. Consequently, the directional parameter of the extended lines* L* and *L*′ in Figure 5 may assume its maximum (at 90°). If we measure similarity (i.e., graduated equality) as inverse to distance and if we establish a distance measure between extended lines that depends on all parameters of the lines parameter space, then* L* and *L*′ in Figure 5 must have* maximum* distance. In other words, their degree of equality is zero, even though they are distinct and incident with* P* and* Q*.

The above observation can be interpreted as* granularity*: if we interpret the extended line* L* in Figure 5 as a* sensor*, then the extended points* P* and* Q* are indiscernible for* L*. Note that in this context grain size is not constant but depends on the line that serves as a* sensor*.

Based on the above-mentioned information, granularity enters Euclid's first postulate, if points and lines have extension: if two extended points* P* and* Q* are* too close* and the extended line* L* is* too broad*, then* P* and* Q* are* indiscernible* for* L*. Since this relation of* indiscernibility* (equality) depends not only on* P* and* Q*, but also on the extended line* L*, which acts as a* sensor*, we denote it by *e*(*P*, *Q*)[*L*], where* L* serves as an additional parameter for the equality of* P* and* Q*.

In [10] the following three boundary conditions to specify a reasonable behavior of *e*(*P*, *Q*)[*L*] were proposed:

(1) If *s*(*L*) ≥ *δ*(*P*, *Q*) + *s*(*P*) + *s*(*Q*), then* P* and* Q* impose no direction constraint on* L* (cf. Figure 5); that is,* P* and* Q* are* indiscernible* for* L* to degree 1: *e*(*P*, *Q*)[*L*] = 1.
(2) If *s*(*L*) < *δ*(*P*, *Q*) + *s*(*P*) + *s*(*Q*), then* P* and* Q* impose some direction constraint on* L* but in general do not fix its location unambiguously. Accordingly, the degree of indiscernibility of* P* and* Q* lies between zero and one: 0 < *e*(*P*, *Q*)[*L*] < 1.
(3) If *s*(*L*) < *δ*(*P*, *Q*) + *s*(*P*) + *s*(*Q*) and *P* = *p*, *Q* = *q*, and *L* = *l* are crisp, then *s*(*L*) = *s*(*P*) = *s*(*Q*) = 0. Consequently,* p* and* q* determine the direction of* l* unambiguously, and all positional tolerance disappears. For this case we demand *e*(*P*, *Q*)[*L*] = 0.

In this paper we are proposing an alternative approach to the one from [10] to model granulated equality.

**Proposition 13 .**

If* fuzzy equivalence relation e*(*P*, *Q*) is defined in (66) and the width *s*(*L*) of* extended line L* is defined in (90), then *e*(*P*, *Q*)[*L*], the degree of* indiscernibility* of* P* and* Q,* could be calculated as follows:

(91) $$\begin{array}{c} e\left(\;P,Q\;\right)\left[\;L\;\right]\equiv e\left(\;P,Q\;\right)\wedge s\left(\;L\;\right), \end{array}$$

and it would satisfy a reasonable behavior, defined in* (1)–(3)*. Here ∧ is* conjunction* operator from Table 1.

**Proof**

From (10), (91), and (66) we have

(92) eP,QL≡eP,Q∧sL=eP,Q+sL2,eP,Q+sL>1,0,eP,Q+sL≤1,

but from (66)

(93) $$\begin{array}{c} e\left(\;P,Q\;\right)=\left\{\begin{array}{cc} \frac{1-p+q}{2}, & p>q,\\ 1, & p=q,\\ \frac{1-q+p}{2}, & p<q; \end{array}\right. \end{array}$$

therefore we have the following:

(1) If* P* and* Q* impose no direction constraint on* L* which means that *s*(*L*) = 1 and *δ*(*P*, *Q*) = 0⇒*e*(*P*, *Q*) = 1, then *e*(*P*, *Q*)[*L*] = 1 (proof of point (1)).
(2) If* P* and* Q* impose some direction constraint on* L* but in general do not fix its location unambiguously, then from (92) and (93) we are getting
  (94) eP,QL=1−p+q+2×sL4,1−p+q2+sL>1,0,1−p−q2+sL≤1,1+sL2,p=q,1−q+p+2×sL4,1−q+p2+sL>1
  ∈(0,1) (proof  of  point  (2)).
(3) If *P* = *p*,   *Q* = *q*, and *L* = *l* are crisp, which means that values of* p* and* q* are either 0 or 1, and since (*L*) = 0, then *e*(*P*, *Q*)[*L*] = 0 (proof of point (3)).

## 4. Fuzzification of Euclid's First Postulate

### 4.1. Euclid's First Postulate Formalization

In previous section we identified and formalized a number of new qualities that enter into Euclid's first postulate, if extended geometric primitives are assumed. We are now in the position of formulating a fuzzified version of Euclid's first postulate. To do this, we first split the postulate which is given as

 “*two distinct points determine a line uniquely*”

into two subsentences:

 “*Given two distinct points, there exists at least one line that passes through them*.”
 “*If more than one line passes through them, then they are equal*.”

These subsentences can be formalized in Boolean predicate logic as follows:

(95) ∀p,q,∃l,  Rincp,l∧Rincq,l,∀p,q,l,m  ¬p=q∧Rincp,l∧Rincq,l∧Rincp,m∧Rincq,m⟶l=m.

A verbatim translation of (95) into the syntax of a fuzzy logic we use yields

(96) infP,Q supL⁡RincP,L∧RincQ,L,

(97) infP,Q,L,M¬eP,Q∧RincP,L∧RincQ,L∧RincP,M∧RincQ,M⟶eL,M,

where* P*,* Q* denote extended points and* L*,* M* denote extended lines. The translated existence property (96) can be adopted as it is, but the translated uniqueness property (97) must be adapted to include* granulated equality* of extended points.

In contrast to the Boolean case, the degree of equality of two given extended points is not constant but depends on the extended line that acts as a* sensor*. Consequently, the term ¬*e*(*P*, *Q*) on the left-hand side of (97) must be replaced by two terms, ¬*e*(*P*, *Q*)[*L*] and ¬*e*(*P*, *Q*)[*M*}, one for each line,* L* and* M*, respectively:
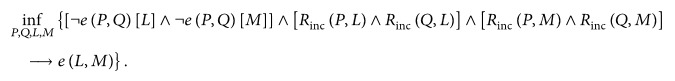
(98) We have to use weak transitivity of graduated equality. For this reason the* discernibility measure* of extended connection $\overset{-}{P}\;\overset{-}{Q}$ between extended points* P* and* Q* must be added into (98):
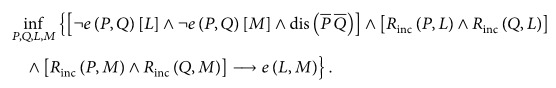
(99)But from (92) we get

(100) ¬eP,QL=2−eP,Q−sL2,eP,Q+sL>1,1,eP,Q+sL≤1,¬eP,QM=2−eP,Q−sM2,eP,Q+sM>1,1,eP,Q+sM≤1.

By using (100) in (99) we get
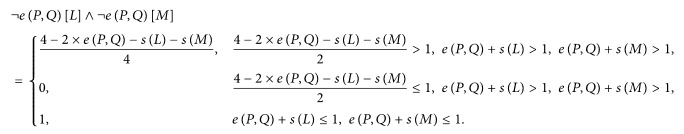
(101)Since from (92) we have [*R*_inc_(*P*, *L*)∧*R*_inc_(*Q*, *L*)]∧[*R*_inc_(*P*, *M*)∧*R*_inc_(*Q*, *M*)] ≡ 1, then (99) could be rewritten as follows:

(102) infP,Q,L,M⁡¬eP,QL∧¬eP,QM∧disP− Q−∧1⟶eL,M.

It means that the “sameness” of extended lines *e*(*L*, *M*) depends on $\left[\neg e(P,Q)[L]\wedge \neg e(P,Q)[M]\wedge \text{dis}\left(\overset{-}{P}\;\overset{-}{Q}\right)\right]$ only and could be calculated by (101) and (79), respectively.

### 4.2. Fuzzy Logical Inference for Euclid's First Postulate

Contrary to the approach proposed in [10], which required a lot of calculations, we suggest using the same fuzzy logic and correspondent logical inference to determine the value of *e*(*L*, *M*). For this purpose let us represent values of *E*(*p*, *q*, *l*, *m*) = ¬*e*(*P*, *Q*)[*L*]∧¬*e*(*P*, *Q*)[*M*] from (101) and $D(p,q)=dis(\overset{-}{P}\;\overset{-}{Q})$ from (79) functions. Note that all values of these functions are lying within certain intervals; that is, *E*(*p*, *q*, *l*, *m*)∈[*E*_min_, *E*_max_] and *D*(*p*, *q*)∈[*D*_min_, *D*_max_]. In our case *E*(*p*, *q*, *l*, *m*)∈[0,1] and *D*(*p*, *q*)∈[0,0.75]. We denote by* E* the* fuzzy set* forming linguistic variables described by triplets of the form $E=\{\langle E_{i},U,\tilde{E}\rangle \},\;\;E_{i}\in T(u),\;\;\forall i\in [0,\mathrm{Card}\;U]$, where *T*_*i*_(*u*) is extended term set of the linguistic variable “*degree of indiscernibility*” from Table 3 and ${\tilde{E}}_{}$ is normal fuzzy set represented by membership function *μ*_*E*_ : *U* → [0,1], where *U* = {0,1, 2,…, 10} is universe set and Card ⁡*U* is power set of the set* U*. We will use the mapping $\alpha :\tilde{E}\to U\;\;\left|u_{i}=Ent\;[(\mathrm{Card}\;U-1)\times E_{i}]\right|\;\;\forall i\in [0,\mathrm{Card}\;U]$, where

(103) $$\begin{array}{c} \tilde{E}=\overset{}{\underset{U}{\int }}\frac{\mu_{E}\left(u\right)}{u}. \end{array}$$

To determine the estimates of the membership function in terms of singletons from (103) in the form *μ*_*E*_(*u*_*i*_)/*u*_*i*_∣∀ *i* ∈ [0, Card ⁡*U*] we propose the following procedure:

(104) μui=1−1Card ⁡U−1×ui−EntCard ⁡U−1×Ei,∀i∈0,Card ⁡U,  ∀Ei∈0,1.

We also denote by* D* the* fuzzy set* forming linguistic variables described by triplets of the form $D=\{\langle D_{j},U,\tilde{D}\rangle \},\;\;D_{j}\in T(u),\;\;\forall j\in [0,\mathrm{Card}\;U]$, where *T*_*j*_(*u*) is extended term set of the linguistic variable “*discernibility measure*” from Table 3 and ${\tilde{D}}_{}$ is normal fuzzy set represented by membership function *μ*_*D*_ : *U* → [0,1].

We will use the mapping $\beta :\tilde{D}\to U\;\;\left|v_{j}=Ent\left[\left(\mathrm{Card}\;U-1\right)\times D_{j}\right]\right|\;\;\forall j\in [0,\mathrm{Card}\;U]$, where

(105) $$\begin{array}{c} \tilde{D}=\overset{}{\underset{U}{\int }}\frac{\mu_{D}\left(u\right)}{u}. \end{array}$$

On the other hand to determine the estimates of the membership function in terms of singletons from (105) in the form *μ*_*D*_(*u*_*j*_)/*u*_*j*_∣∀ *j* ∈ [0, Card ⁡*U*] we propose the following procedure:

(106) μuj=1−1Card ⁡U−1×uj−EntCard ⁡U−1×Dj0.75,∀j∈0,Card ⁡U,  ∀Dj∈0,0.75.

Let us represent *e*(*L*, *M*) as a* fuzzy set* $\tilde{S}$, forming linguistic variables described by triplets of the form $S=\{\langle S_{k},V,\tilde{S}\rangle \}$, *S*_*k*_ ∈ *T*(*v*), ∀*k* ∈ [0, Card ⁡*V*], where *T*_*k*_(*V*) is extended term set of the linguistic variable “extended lines sameness” from Table 3. $\tilde{S}$ is normal fuzzy set represented by membership function *μ*_*S*_ : *V* → [0,1], where *V* = {0,1, 2,…, 10} is universe set and Card ⁡*V* is power set of the set *V*. We will use the mapping $\gamma :\tilde{S}\to V\left|v_{k}=Ent\;[(\mathrm{Card}\;V-1)\times S_{k}]\right|\;\;\forall k\in [0,\mathrm{Card}\;V]$, where

(107) $$\begin{array}{c} \tilde{S}=\overset{}{\underset{V}{\int }}\frac{\mu_{s}\left(v\right)}{v}. \end{array}$$

Again to determine the estimates of the membership function in terms of singletons from (107) in the form *μ*_*S*_(*w*_*k*_)/*v*_*k*_∣∀ *k* ∈ [0, Card ⁡*V*], we propose the following procedure:

(108) μvk=1−1Card ⁡V−1×vk−EntCard ⁡V−1×Sk,∀k∈0,Card ⁡V,  ∀Sk∈0,1.

To get estimates of values of *e*(*L*, *M*) or “*extended lines sameness,”* represented by fuzzy set $\tilde{S}$ from (107) given the values of *E*(*p*, *q*, *l*, *m*) or “*degree of indiscernibility*” and *D*(*p*, *q*) or “*discernibility measure*” represented by fuzzy sets $\tilde{E}$ from (103) and $\tilde{D}$ from (105), respectively, we will use a* Fuzzy Conditional Inference Rule*, formulated by means of* “common sense”* as the following conditional clause:

(109) P=“IF S  is  P1AND  D  is  P2,THEN E  is  Q”.

In words we use fuzzy conditional inference of the following type:

(110) $$\begin{array}{c} \begin{array}{c} \text{Ant1: If }s\text{ is }P1\text{ and }d\text{ is }P2\text{ then }e\text{ is }Q\\ \text{Ant2: }s\text{ is }P1^{\prime }\text{ and }d\text{ is }P2^{\prime }\\ \text{Cons: }e\text{ is }Q^{\prime }, \end{array} \end{array}$$

where *P*1, *P*1′, *P*2, *P*2′⊆*U* and *Q*, *Q*′⊆*V*.

Now for fuzzy sets (103), (105), and (107) a* binary relationship* for the fuzzy conditional proposition of the type of (109) and (110) for fuzzy logic we use so far is defined as

(111) RA1s,d,A2e=P1∩P2×U⟶V×Q=∫U×VμP1uu,v∧μP2uu,v⟶∫U×VμQvu,v=∫U×VμP1u∧μP2u⟶μQvu,v.

Given (11), expression (111) looks like

(112) $$\begin{array}{c} \left[\;\mu_{P1}\left(\;u\;\right)\wedge \mu_{P2}\left(\;u\;\right)\;\right]⟶\mu_{Q}\left(\;v\;\right)=\left\{\begin{array}{cc} \frac{1-\left[\mu_{P1}\left(u\right)\wedge \mu_{P2}\left(u\right)\right]+\mu_{Q}\left(v\right)}{2}, & \left[\mu_{P1}\left(u\right)\wedge \mu_{P2}\left(u\right)\right]>\mu_{Q}\left(v\right),\\ 1, & \left[\mu_{P1}\left(u\right)\wedge \mu_{P2}\left(u\right)\right]\leq \mu_{Q}\left(v\right), \end{array}\right. \end{array}$$

where [*μ*_*P*1_(*u*)∧*μ*_*P*2_(*u*)] is min⁡[*μ*_*P*1_(*u*), *μ*_*P*2_(*u*)]. It is well known that given a* unary relationship R*(*A*_1_(*s*, *d*)) = *P*1′∩*P*2′ one can obtain the consequence *R*(*A*_2_(*e*)) by applying compositional rule of inference (CRI) to *R*(*A*_1_(*s*, *d*)) and *R*(*A*_1_(*s*, *d*), *A*_2_(*e*)) of type (111):

(113) RA2e=P1′∩P2′∘RA1s,d,A2e=∫UμP1′u∧μP2′uu∘∫U×VμP1u∧μP2u⟶μQvu,v=∫V⋃u∈UμP1′u∧μP2′u∧μP1u∧μP2u⟶μQvv.

But for practical purposes we will use another* Fuzzy Conditional Rule* (FCR):

(114) RA1s,d,A2e=P×V⟶U×Q∩¬P×V⟶U×¬Q=∫U×VμPu⟶μQv∧1−μPu⟶1−μQvu,v,

where *P* = *P*1∩*P*2 and

(115) RA1s,d,A2e=μPu⟶μQv∧1−μPu⟶1−μQv=1−μPu+μQv2,μPu>μQv,1,μPu=μQv,1−μQv+μPu2,μPu<μQv.

The FCR from (115) gives more reliable results.

### 4.3. Example

To build a binary relationship matrix of type (114) we use a conditional clause of type (109):

(116) P=“IF S  is  “lowest”  AND  D  is  “highest”,THEN E  is  “nothing  in  common””.

Note that values* “lowest*” of a linguistic variable “*degree of indiscernibility,*” “*highest”* of a linguistic variable “*discernibility measure,*” and “*nothing in common*” of a linguistic variable “*extended lines sameness”* have come from Table 3. This particular knowledge is based on common sense and represents a simple human perception of geometrical facts. It is worth mentioning that (116) might be equivalently replaced by the following knowledge statement:

(117) P=“IF S  is  “highest”AND  D  is  “lowest”,  THEN E  is  “the  same””.

To build membership functions for fuzzy sets* S*,* D,* and* E* we use (104), (106), and (108), respectively.

In (116) the membership functions for fuzzy set* S* (for instance) would look like

(118) μs“lowest”=10+0.91+0.82+0.73+0.64+0.55+0.46+0.37+0.28+0.19+010,

which are the same membership functions we use for fuzzy sets* D* and* E.*

From (115) we have *R*(*A*_1_(*s*, *d*), *A*_2_(*e*)) from Table 4.

Suppose from (101) a current estimate of *E*(*p*, *q*, *l*, *m*) = 0.6 and from (79) *D*(*p*, *q*) = 0.25. By using (104) and (106), respectively, we got (see Table 3)

(119) μE“bit  higher  than  average”=0.40+0.51+0.62+0.73+0.84+0.95+16+0.97+0.88+0.79+0.610,μD“pretty  high”=0.70+0.81+0.92+13+0.94+0.85+0.76+0.67+0.58+0.49+0.310.

It is apparent that

(120) RA1s′,d′μEu∧μDu=0.40+0.51+0.62+0.73+0.84+0.85+0.76+0.67+0.58+0.49+0.310.

By applying compositional rule of inference (CRI) to *R*(*A*_1_(*s*′, *d*′)) and *R*(*A*_1_(*s*, *d*), *A*_2_(*e*)) from Table 4 *R*(*A*_2_(*e*′)) = *R*(*A*_1_(*s*′, *d*′))∘*R*(*A*_1_(*s*, *d*), *A*_2_(*e*) we got the following:  *R*(*A*_2_(*e*′)) = 0.4/0 + 0.5/1 + 0.6/2 + 0.7/3 + 0.8/4 + 0.8/5 + 0.7/6 + 0.6/7 + 0.5/8 + 0.4/9 + 0.3/10.

Since the maximum value of membership function of the consequence (for two singletons 0.8/4 and 0.8/5) is given as

(121) $$\begin{array}{c} R\left(\;A_{2}\left(\;e^{\prime }\;\right)\;\right)=0.8, \end{array}$$

then it is obvious that the value of fuzzy set* S* is lying between terms* “almost average distance”* and* “average distance”* (see Table 3), which means that

(122) $$\begin{array}{c} e\left(\;L,M\;\right)\in \left[\;0.5,0.6\;\right]. \end{array}$$

## 5. Conclusion

In [1–4] it was shown that straight forward interpretations of the connection of extended points do not satisfy the incidence axioms of Euclidean geometry in a strict sense. We formalized the axiom system of Boolean-Euclidean geometry by the language of the fuzzy logic [5]. We also addressed fuzzification of Euclid's first postulate by using the same fuzzy logic. Fuzzy equivalence relation* “extended lines sameness”* is introduced. For its approximation we use fuzzy conditional inference, which is based on proposed fuzzy* “degree of indiscernibility”* and* “discernibility measure”* of extended points.

## Figures and Tables

**Table 1:** 

Name	Designation	Value
Tautology	*P* ^*I*^	1

Controversy	*P* ^*O*^	0

Negation	¬*P*	1 − *P*

Disjunction	*P*∨*Q*	$\left\{\begin{array}{cc} \frac{p+q}{2}, & p+q<1,\\ 1, & p+q\geq 1 \end{array}\right.$

Conjunction	*P*∧*Q*	$\left\{\begin{array}{cc} \frac{p+q}{2}, & p+q>1,\\ 0, & p+q\leq 1 \end{array}\right.$

Implication	*P* → *Q*	$\left\{\begin{array}{cc} \frac{1-p+q}{2}, & p>q,\\ 1, & p\leq q \end{array}\right.$

Equivalence	*P*↔*Q*	$\left\{\begin{array}{cc} \frac{1-p+q}{2}, & p>q,\\ 1, & p=q,\\ \frac{1-q+p}{2}, & p<q \end{array}\right.$

Pierce arrow	*P* ↓ *Q*	$\left\{\begin{array}{cc} 1-\frac{p+q}{2}, & p+q<1,\\ 0, & p+q\geq 1 \end{array}\right.$

Shaffer stroke	*P*↑*Q*	$\left\{\begin{array}{cc} 1-\frac{p+q}{2}, & p+q>1,\\ 1, & p+q\leq 1 \end{array}\right.$

**Table 2:** 

*p* → *q*	0	0.1	0.2	0.3	0.4	0.5	0.6	0.7	0.8	0.9	1
0	1	1	1	1	1	1	1	1	1	1	1
0.1	0.45	1	1	1	1	1	1	1	1	1	1
0.2	0.4	0.45	1	1	1	1	1	1	1	1	1
0.3	0.35	0.4	0.45	1	1	1	1	1	1	1	1
0.4	0.3	0.35	0.4	0.45	1	1	1	1	1	1	1
0.5	0.25	0.3	0.35	0.4	0.45	1	1	1	1	1	1
0.6	0.2	0.25	0.3	0.35	0.4	0.45	1	1	1	1	1
0.7	0.15	0.2	0.25	0.3	0.35	0.4	0.45	1	1	1	1
0.8	0.1	0.15	0.2	0.25	0.3	0.35	0.4	0.45	1	1	1
0.9	0.05	0.1	0.15	0.2	0.25	0.3	0.35	0.4	0.45	1	1
1	0	0.05	0.1	0.15	0.2	0.25	0.3	0.35	0.4	0.45	1

**Table 3:** 

Value of variable			*u*_*i*_, *v*_*j*_ ∈ *U*, *v*_*k*_ ∈ *V* ∀*i*, *j*, *k* ∈ [0,10]
“Degree of indiscernibility”	“Discernibility measure”	“Extended lines sameness”	
Lowest	Highest	Nothing in common	0
Very low	Almost highest	Very far	1
Low	High	Far	2
Bit higher than low	Pretty high	Bit closer than far	3
Almost average	Bit higher than average	Almost average distance	4
Average	Average	Average	5
Bit higher than average	Almost average	Bit closer than average	6
Pretty high	Bit higher than low	Pretty close	7
High	Low	Close	8
Almost highest	Very low	Almost the same	9
Highest	Lowest	The same	10

**Table 4:** 

	1	0.9	0.8	0.7	0.6	0.5	0.4	0.3	0.2	0.1	0
1	1	0.45	0.4	0.35	0.3	0.25	0.2	0.15	0.1	0.05	0
0.9	0.45	1	0.45	0.4	0.35	0.3	0.25	0.2	0.15	0.1	0.05
0.8	0.4	0.45	1	0.45	0.4	0.35	0.3	0.25	0.2	0.15	0.1
0.7	0.35	0.4	0.45	1	0.45	0.4	0.35	0.3	0.25	0.2	0.15
0.6	0.3	0.35	0.4	0.45	1	0.45	0.4	0.35	0.3	0.25	0.2
0.5	0.25	0.3	0.35	0.4	0.45	1	0.45	0.4	0.35	0.3	0.25
0.4	0.2	0.25	0.3	0.35	0.4	0.45	1	0.45	0.4	0.35	0.3
0.3	0.15	0.2	0.25	0.3	0.35	0.4	0.45	1	0.45	0.4	0.35
0.2	0.1	0.15	0.2	0.25	0.3	0.35	0.4	0.45	1	0.45	0.4
0.1	0.05	0.1	0.15	0.2	0.25	0.3	0.35	0.4	0.45	1	0.45
0	0	0.05	0.1	0.15	0.2	0.25	0.3	0.35	0.4	0.45	0.1
